# Long-term adherence to inhaled corticosteroids and asthma control in adult-onset asthma

**DOI:** 10.1183/23120541.00715-2020

**Published:** 2021-02-08

**Authors:** Iida Vähätalo, Hannu Kankaanranta, Leena E. Tuomisto, Onni Niemelä, Lauri Lehtimäki, Pinja Ilmarinen

**Affiliations:** 1 Dept of Respiratory Medicine, Seinäjoki Central Hospital, Seinäjoki, Finland; 2 Tampere University Respiratory Research Group, Faculty of Medicine and Health Technology, Tampere University, Tampere, Finland; 3 Krefting Research Centre, Institute of Medicine, Dept of Internal Medicine and Clinical Nutrition, University of Gothenburg, Gothenburg, Sweden; 4 Dept of Laboratory Medicine, Seinäjoki Central Hospital, Seinäjoki, Finland; 5 Allergy Centre, Tampere University Hospital, Tampere, Finland

## Abstract

**Background:**

In short-term studies, poor adherence to inhaled corticosteroids (ICS) has been associated with worse asthma control, but the association of long-term adherence and disease control remains unclear.

**Objective:**

To assess the relationship between 12-year adherence to ICS and asthma control in patients with adult-onset asthma.

**Methods:**

As part of the Seinäjoki Adult Asthma Study, 181 patients with clinically confirmed new-onset adult asthma and regular ICS medication were followed-up for 12 years. Adherence (%) to ICS was assessed individually ((µg dispensed/µg prescribed)×100) during the follow-up. Asthma control was evaluated after 12 years of treatment according to the Global Initiative for Asthma 2010 guideline.

**Results:**

Asthma was controlled in 31% and not controlled (partly controlled or uncontrolled) in 69% of the patients. Patients with not-controlled asthma were more often male, older, nonatopic and used higher doses of ICS than those with controlled disease. The mean±sd 12-year adherence to ICS was 63±38% in patients with controlled asthma and 76±40% in patients with not-controlled disease (p=0.042). Among patients with not-controlled asthma, those with lower 12-year adherence (<80%) had more rapid decline in forced expiratory volume in 1 s (−47 mL·year^−1^) compared to patients with better adherence (≥80%) (−40 mL·year^−1^) (p=0.024). In contrast, this relationship was not seen in patients with controlled asthma.

**Conclusions:**

In adult-onset asthma, patients with not-controlled disease showed better 12-year adherence to ICS treatment than those with controlled asthma. In not-controlled disease, adherence <80% was associated with more rapid lung function decline, underscoring the importance of early recognition of such patients in routine clinical practice.

## Abstract 2

**Patients with not-controlled asthma and poor adherence show increased FEV_1_ decline. Special emphasis should be placed on ICS adherence in subjects who do not have controlled asthma, as they seem to be at higher risk of developing fixed airway obstruction.** https://bit.ly/2LOXL4f

## Introduction

Successful asthma treatment plays a pivotal role in preventing exacerbations, enhancing patients’ quality of life and decreasing healthcare costs [1]. Asthma often remains poorly controlled despite effective pharmacological treatment strategies [2–4] and current guidelines emphasise the importance of finding out the reason behind not-controlled asthma in each patient [5]. Age of asthma onset has been shown to differentiate the phenotypes of asthma [6, 7], but very little information exists on the disease control characteristics of the late-onset asthma phenotype [3].

To gain optimal benefits from pharmacotherapy, patients should be adherent to treatment, which has been shown to be often suboptimal [1, 8]. Only two studies so far have evaluated long-term adherence to inhaled corticosteroids (ICS): the Childhood Asthma Management Program (CAMP) (4-year follow-up) [9] and the Seinäjoki Adult Asthma Study (SAAS) (12-year follow-up) [8]. In these studies, mean adherence to ICS was 52% and 69%, respectively. Previous studies assessing asthma control and adherence have been either cross-sectional or with short follow-up [10–18]. In addition, the evaluation of adherence and asthma control has mostly been questionnaire-based and information concerning diagnostic criteria, duration and age of onset of asthma are often missing, potentially influencing the results [4, 13, 15]. Poor asthma control has been associated with higher risk of exacerbations, lower quality of life and increased healthcare use [2, 4, 10, 19]. Previous studies have suggested that suboptimal adherence to pharmacological therapy impairs asthma control [4, 10–13, 20]. In contrast, a recent study identified that patients with uncontrolled asthma were more adherent to ICS treatment [21]. However, the adherence was determined from prescriptions issued, reflecting the physician's prescription manners, not the adherence of the patient. It should be noted that in previous studies medication possession ratio (MPR) and proportion of days covered (PDC) formulas have been used regularly for estimating adherence [1, 21]. Unfortunately, the data used in these formulas usually lack detail, such as did patients have continuous prescription for ICS and how were dose ranges and single maintenance and reliever therapy regarded, all being relevant issues in the treatment of asthma.

Inadequate use of preventer medication is suggested to be related to decline in lung function, but there are no data on the association between long-term adherence and lung function decline stratified by asthma control. An Australian study [22] found accelerated lung function in patients not taking adequate preventer therapy. Furthermore, in previous short-term (1-year) follow-up study conducted in the United Kingdom [23], patients with difficult-to-control asthma and suboptimal ICS adherence had reduced forced expiratory volume in 1 s (FEV_1_). In our recent study, poorer 12-year adherence was related to lung function decline in the long-term, but patients with good adherence used more add-on drugs, oral corticosteroid courses, had more hospital days and used more healthcare services, *i.e.* had features suggesting not-controlled asthma [8]. Thus, we hypothesised that not-controlled asthma is not a direct consequence of poor adherence and that lung function decline does not depend on poor adherence only, but may be affected by asthma control. Hence this study aimed to assess the relationship between 12-year adherence to ICS and asthma control in patients with adult-onset asthma, especially concentrating on whether the effect of poor adherence on lung function decline is affected by asthma control. In this study, we used full-coverage dispensing data and information on prescribed ICS, offering the possibility to assess real-life adherence based on dispensed and prescribed amounts of ICS [8, 24].

## Methods

### Study design and patients

The current study is part of SAAS, which is a prospective 12-year follow-up study of patients with diagnosis of new-onset adult asthma. All new adult (age ≥15 years) patients in Seinäjoki Central Hospital were included during the period 1999–2002. Diagnostic criteria, inclusion and exclusion criteria have been reported previously [25] (supplementary eTable 1). Patients with comorbidities or smoking history were not excluded. Study participants gave written informed consent to the study protocol approved by the ethics committee of Tampere University Hospital (Tampere, Finland).

**TABLE 1:** Characteristics of asthma patients at 12 years after diagnosis according to their level of asthma control (n=181)^#^

	**Controlled**	**Not-controlled**	**p-value**
**Patients**	56	125	
**Age years**	56±14.6	61±12.4	0.011^++^
**Female**	41 (73.2)	67 (53.6)	0.014^§§^
**BMI kg·m^−2^**	27.6±3.8	29.1±6.0	0.079^++^
**Smokers (including ex-smokers)**	18 (32.1)	73 (58.4)	0.001^§§^
**Smoking history pack-years**	7 (2–12)	20 (10–32)	<0.001^ƒƒ^
**≥10** **pack-years and post-BD FEV_1_/FVC <0.7**^¶^	4 (7.1)	29 (23.4)	0.011^§§^
**Pre-BD FEV_1_% pred**	92 (86–99)	82 (70–93)	<0.001^ƒƒ^
**Pre-BD FEV_1_/FVC**	0.75 (0.70–0.79)	0.73 (0.64–0.78)	0.016^ƒƒ^
**Post-BD FEV_1_% pred**	96 (90–101)	84 (75–96)	<0.001^ƒƒ^
**Post-BD FEV_1_/FVC**	0.77 (0.73–0.83)	0.73 (0.65–0.79)	0.002^ƒƒ^
**Blood eosinophils ×10^9^·L^−1^**	0.17 (0.12–0.28)	0.18 (0.09–0.27)	0.353^ƒƒ^
**Total IgE kU·L^−1^**	51 (28–161)	71 (24–172)	0.617^ƒƒ^
***F*_eNO_ ppb**	12 (6–19)	10 (5–18)	0.392^ƒƒ^
**Blood neutrophils ×10^9^·L^−1^**	3.7 (3.0–4.6)	3.9 (2.9–4.9)	0.522^ƒƒ^
**Prescribed daily dose of ICS µg BDP**	751 (502–939)	838 (664–1023)	0.014^ƒƒ^
**Dispensed daily dose of ICS µg BDP**	411 (246–625)	602 (354–838)	0.002^ƒƒ^
**Daily SABA**^**+**^	2 (3.6)^###^	19 (15.2)	0.024^§§^
**Daily LABA**^**+**^	18 (32.1)	77 (61.6)	<0.001^§§^
**Self-reported use of oral corticosteroid courses for asthma^§^**	12 (21.4)	48 (39.0)	0.06^§§^
**Dispensed oral corticosteroid for asthma per year mg^ƒ^**	44 (0–127)	92 (0–240)	0.013^ƒƒ^
**Comorbidities**	1 (0–2)	1 (0–3)	0.057^ƒƒ^
**Co-medications (nonrespiratory)**	1 (0–4)	2 (0–4)	0.124^ƒƒ^
**AQ20 score**	2 (0–4)	6 (3–9)	<0.001^ƒƒ^
**ACT score**	24 (22–25)	20 (17–23)	<0.001^ƒƒ^
**Asthma-related visits to healthcare^§,##^**	12 (6–19)	16 (10–26)	0.014^ƒƒ^
**Atopy^¶¶^**	27 (50.9)	34 (30.6)	0.016^§§^
**≥1 hospital in-patient periods, asthma-related (unplanned)^§^**	1 (1.8)	15 (12.0)	0.024^§§^

Data are presented as n, mean±sd, n (%) or median (interquartile range), unless otherwise stated. BMI: body mass index; BD: bronchodilator; FEV_1_: forced expiratory volume in 1 s; FVC: forced vital capacity; IgE: immunoglobulin E; *F*_eNO_: exhaled nitric oxide fraction; ICS: inhaled corticosteroid; BDP: beclomethasone dipropionate equivalents; SABA: short-acting β_2_-agonist; LABA: long-acting β_2_-agonist; AQ20: Airways Questionnaire 20; ACT: Asthma Control Test. ^#^: the results of lung function and inflammatory parameters have been published previously in patients with controlled, partially controlled and uncontrolled asthma [3]; ^¶^: baseline ≥10 pack-years and post-BD FEV_1_/FVC ratio <0.7 in patients with controlled asthma (n=2, 3.6%) and patients with not-controlled asthma (n=13, 10.7%) (p=0.150); ^+^: self-reported daily use; ^§^: examined during the whole 12-year follow-up period; ^ƒ^: values obtained from the Finnish Social Insurance Institution and were divided by the years of follow-up; ^##^: all respiratory-related scheduled and unscheduled contacts with healthcare due to asthma; ^¶¶^: defined as ≥1 positive response (≥3 mm) in skin prick test towards common aeroallergens [31]; ^++^: independent samples t-test; ^§§^: Fisher's exact test; ^ƒƒ^: independent samples Mann–Whitney U-test; ^###^: these two patients were not dispensed SABA in the year when asthma control was determined and therefore they were considered to belong to the group of controlled patients. However, they self-reported daily use of SABA and more SABA was dispensed in preceding years of the follow-up.

**FIGURE 1:**
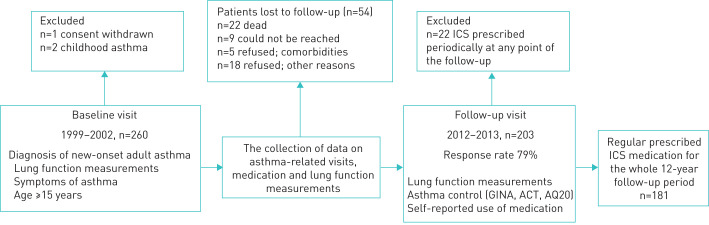
Flow-chart of the study. ICS: inhaled corticosteroids; GINA: Global Initiative for Asthma; ACT: Asthma Control Test; AQ20: Airway Questionnaire 20.

The study was divided into two parts: baseline visit and 12-year follow-up visit (figure 1). At the baseline visit, data were collected on symptoms, lung function and demographics, as described previously [25]. Furthermore, regular ICS medication was prescribed and each patient received asthma education, advice on correct inhaler use and self-management instructions according to the Finnish asthma programme [26]. From the original cohort of 257 patients, 203 (79%) returned for the 12-year follow-up visit in which asthma control, medication and lung function were evaluated (supplementary material). All asthma-related visits and medication information were collected for the whole 12-year follow-up period from medical records [24]. To ensure that the study population included only patients with regular ICS medication, we excluded patients for whom ICS was prescribed only periodically (often Global Initiative for Asthma (GINA) step 1 and ICS use during pollen season) at any point of the follow-up (figure 1).

### Asthma control and lung function

Asthma control was defined according to GINA 2010 [27] and “not-controlled” included both partially and uncontrolled asthma (supplementary material). Lung function measurement points were 1) baseline (diagnosis), 2) the maximum lung function (max_0–2.5_) during the first 2.5 years after diagnosis (after start of therapy) and 3) 12-year follow-up visit (supplementary material). Decline in lung function during the 12-year follow-up period was defined as change in pre-bronchodilator FEV_1_ from max_0–2.5_ to the 12-year time point.

### Assessment of adherence

The prescribed ICS dose in each patient for the 12-year period was calculated based on medication records, as described previously [24]. Briefly, we converted all prescribed ICS doses (ICS in both single and combination inhalers) to beclomethasone dipropionate equivalents, and based on that information, calculated annual prescribed ICS medication for each patient. The dispensed ICS doses were obtained from the Finnish Social Insurance Institution which records all medication purchased from any Finnish pharmacy. All drug and dose changes were taken into account individually. In the case of ranged doses prescribed (*e.g.* one or two puffs twice daily), we interpreted that patients were adherent when the minimum ICS doses were dispensed. Adherence to ICS was determined as described recently [8], consisting of initiation, implementation and persistence (supplementary material). The 12-year adherence was calculated by comparing cumulative dispensed doses of ICS (µg) to cumulative prescribed doses of ICS (µg) and annual adherence by comparing yearly dispensed doses of ICS (µg) to yearly prescribed doses of ICS (µg). This adherence calculation combines elements from both MPR and PCD formulas (supplementary material) [8, 28] and we estimated the time-variance of the adherence according to a recent publication [29].

### Statistical analyses

The results are shown as mean±sd or median (interquartile range), but annual adherence is represented as mean±sem for clarity. Comparison of groups with ≥80% or <80% adherence to ICS were analysed by using independent samples t-test and Mann–Whitney U-test for normally and non-normally distributed continuous variables, respectively, and Pearson's Chi-squared or Fisher's exact test for categorical variables. To analyse differences in annual adherence over the 12-year period between controlled and not-controlled patients, annual adherence was plotted against time for individual patients, and mean area under curve values were compared using an independent samples t-test. A multivariable binary logistic regression analysis was performed to analyse factors associated with not-controlled asthma. A multiple linear regression analysis was performed to analyse factors associated with FEV_1_ decline, as described previously [30]. The correlation matrix was analysed and covariates not strongly correlated (r<0.7) (age, sex, body mass index (BMI) at follow-up, exhaled nitric oxide fraction (*F*_eNO_) >20 ppb, ≥10 pack-years at follow-up, change (Δ) in FEV_1_ (baseline−max_0–2.5_) and average 12-year adherence (<80%) to ICS) were included in the analysis and outliers were removed to ensure homoscedasticity (supplementary material). A p-value <0.05 was regarded as statistically significant. Statistical analyses were performed using SPSS statistics software (version 24; IBM, Armonk, NY, USA) and GraphPad Prism (version 7.03; GraphPad, La Jolla, CA, USA).

## Results

### Patient characteristics

The majority of the study patients were female (60%), the average age was 59±13 years at the follow-up visit and half of the patients were current or ex-smokers (supplementary eTable 2). At the follow-up visit patients had higher BMI, better lung function, lower blood eosinophil counts and fewer symptoms (Asthma Questionnaire 20) compared to the baseline visit (supplementary eTable 2).

**TABLE 2:** Characteristics of patients with not-controlled asthma at 12 years after diagnosis according to their level of 12-year adherence (n=125)

	**Good adherence (≥80%)**	**Poor adherence (<80%)**	**p-value**
**Patients**	61	64	
**Age years**	62±12	60±13	0.242^ƒ^
**Female**	36 (59.0)	31 (48.4)	0.283^##^
**BMI kg·m^−2^**	28.4 (24.6–32.5)	28.5 (24.5–32.3)	0.286^¶¶^
**Smokers (including ex-smokers)**	35 (57.4)	38 (59.4)	0.857^##^
**Smoking history pack-years**	19 (9–34)	20 (12–30)	0.977^¶¶^
**Pre-BD FEV_1_ % pred**	84 (71–99)	80 (70–90)	0.200^¶¶^
**Pre-BD FEV_1_/FVC**	0.73 (0.65–0.78)	0.72 (0.63–0.78)	0.797^¶¶^
**Post-BD FEV_1_% pred**	84 (75–99)	84 (75–92)	0.386^¶¶^
**Post-BD FEV_1_/FVC**	0.73 (0.66–0.79)	0.73 (0.65–0.80)	0.888^¶¶^
**Blood eosinophils ×10^9^·L^−1^**	0.15 (0.08–0.25)	0.19 (0.10–0.29)	0.118^¶¶^
**Total IgE kU·L^−1^**	61 (23–138)	79 (29–197)	0.140^¶¶^
**Blood neutrophils ×10^9^·L^−1^**	4.2 (3.4–5.2)	3.5 (2.7–4.6)	0.022^¶¶^
**Prescribed daily dose of ICS µg BDP**	841 (704–1062)	834 (642–995)	0.412^¶¶^
**Dispensed daily dose of ICS µg BDP**	831 (728–1+)	375 (210–520)	<0.001^¶¶^
**Daily SABA^#^**	13 (21.3)	6 (9.4)	0.082^##^
**Daily LABA^#^**	46 (75.4)	31 (48.4)	0.003^##^
**Daily LTRA^#^**	16 (26.2)	6 (9.5)	0.019^##^
**Self-reported use of oral corticosteroid courses for asthma^¶^**	26 (44.1)	22 (34.4)	0.355^##^
**Dispensed oral corticosteroid for asthma per year mg**^**+**^	125 (10–273)	70 (0–193)	0.176^¶¶^
**Co-medications (non-respiratory)**	2 (1–5)	2 (0–4)	0.116^¶¶^
**AQ20 score**	6 (3.5–8)	5.5 (2–10)	0.822^¶¶^
**ACT score**	21 (18–23)	20 (16–23)	0.795^¶¶^
**Allergy and/or rhinitis**	45 (73.8)	46 (71.9)	0.843^##^
**Asthma-related visits to healthcare^¶,§^**	19 (13–28)	13 (9–22)	0.005^¶¶^

Data are presented as n, mean±sd, n (%) or median (interquartile range), unless otherwise stated. BMI: body mass index; BD: bronchodilator; FEV_1_: forced expiratory volume in 1 s; FVC: forced vital capacity; IgE: immunoglobulin E; ICS: inhaled corticosteroid; BDP: beclomethasone dipropionate equivalents; SABA: short-acting β_2_-agonist; LABA: long-acting β_2_-agonist; LTRA: leukotriene receptor antagonist; AQ20: Airways Questionnaire 20; ACT: Asthma Control Test. ^#^: self-reported daily use; ^¶^: examined during the whole 12-year follow-up period; ^+^: dispensed doses of oral corticosteroids (mg) were obtained from the Finnish Social Insurance Institution and were divided by the years of follow-up; ^§^: all respiratory-related scheduled and unscheduled contacts with healthcare due to asthma; ^ƒ^: independent samples t-test; ^##^: Fisher's exact test; ^¶¶^: independent samples Mann–Whitney U-test.

### Asthma control

At the 12-year follow-up visit, asthma control was evaluated and the patients were divided into two groups: controlled (n=56) and not controlled (n=125). Group characteristics are shown in table 1. Patients with not-controlled asthma were more often male, older and were prescribed higher doses of ICS than patients with controlled asthma. As reported previously, lung function was better and smoking was less common in patients with controlled asthma *versus* not-controlled asthma [3]. Patients with not-controlled asthma used more daily add-on drugs, had more days in hospital and were dispensed higher doses of oral corticosteroids (table 1). In addition, patients with not-controlled asthma were less often atopic and had a higher number of asthma-related contacts with healthcare. No difference was found in inflammatory parameters.

### Adherence and asthma control

**FIGURE 2:**
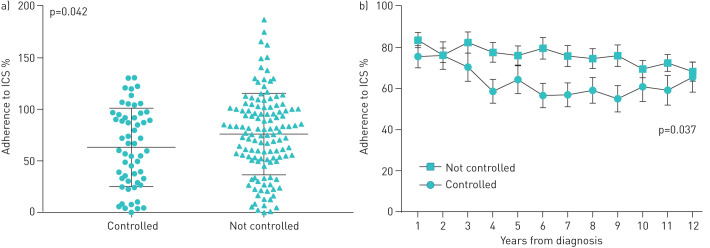
Long-term adherence to inhaled corticosteroids (ICS) in patients with controlled and not-controlled asthma. a) The average 12-year adherence to ICS in study subgroups (mean±sd). Adherence >100% means that patients were dispensed more than a regular individually prescribed minimum dose of ICS. b) The average annual adherence (mean±sem) in patients with controlled and not-controlled asthma during the 12-year follow-up period. p-value represents difference in annual ICS adherence between not-controlled and controlled patients as defined by area under the curve method and independent-samples t-test. Significant difference was also seen when patients with COPD were excluded from the analyses (a) p=0.021 and b) p=0.019).

The mean±sd 12-year adherence to ICS was 63±38% in patients with controlled asthma and 76±40% in patients with not-controlled disease (p=0.042) (figure 2a). Patients with not-controlled asthma had significantly higher adherence (p=0.037) compared to patients with controlled asthma in the whole 12-year study period (figure 2b). Furthermore, 34% of the study patients had not-controlled asthma despite having ≥80% adherence to ICS treatment during 12-year follow-up (table 2). The association between ≥80% adherence and not-controlled asthma remained in binary logistic regression analysis adjusting for age ≥60 years, BMI ≥30 kg·m^−2^, sex, COPD and rhinitis. When evaluating long-term ICS use, it was found that 76.8% of the patients with not-controlled asthma and 60.7% of the patients with controlled asthma were >50% adherent to their ICS treatment each year during the 12-year follow-up (p=0.032).

### Not-controlled asthma

A large variation in the ICS adherence was found in the not-controlled asthma group. Therefore, we considered that there may be two different groups of patients with suboptimal asthma control: 1) those having not-controlled asthma due to low adherence to ICS; and 2) those having not-controlled asthma despite good adherence to ICS. To see whether clinical differences exist between these groups, we evaluated asthma-related parameters in patients having not-controlled asthma and ≥80% or <80% 12-year adherence (table 2 and supplementary eTable3). The patients having not-controlled asthma and ≥80% adherence had a higher number of asthma-related contacts with healthcare, higher blood neutrophil count and used more often long-acting β_2_-agonists (LABA) or leukotriene receptor antagonists (table 2).

### Controlled asthma

Assessment of patients with good asthma control revealed that patients with ≥80% adherence had lower BMI, higher total immunoglobulin E and peripheral blood neutrophil counts and lower FEV_1_ reversibility (mL) than patients with <80% adherence and controlled asthma (table 3 and supplementary eTable 4). In addition, patients with controlled asthma and ≥80% adherence reported using oral corticosteroids more often and had tendency to increased asthma-related visits to healthcare compared to <80% adherent patients.

**TABLE 3:** Characteristics of patients with controlled asthma at 12 years after diagnosis according to their level of 12-year adherence (n=56)

	**Good adherence (≥80%)**	**Poor adherence (<80%)**	**p-value**
**Patients**	21	35	
**Age years**	58±11	54±16	0.266^ƒ^
**Female**	16 (76.2)	25 (71.4)	0.764^##^
**BMI kg·m^−2^**	26.3 (3.4)	28.3 (3.8)	0.045^ƒ^
**Smokers (including ex-smokers)**	5 (23.8)	13 (37.1)	0.382^##^
**Smoking history pack-years**	10 (3.7–14.8)	5.3 (1.3–9.3)	0.383^¶¶^
**Pre-BD FEV_1_ % pred**	91 (86–100)	92 (86–98)	0.939^¶¶^
**Pre-BD FEV_1_/FVC**	0.74 (0.68–0.80)	0.75 (0.71–0.79)	0.460^¶¶^
**Post-BD FEV_1_% pred**	96 (90–100)	96 (91–102)	0.826^¶¶^
**Post-BD FEV_1_/FVC**	0.75 (0.71–0.82)	0.78 (0.73–0.83)	0.285^¶¶^
**Blood eosinophils ×10^9^·L^−1^**	0.25 (0.13–0.37)	0.15 (0.11–0.26)	0.095^¶¶^
**Total IgE kU·L^−1^**	93 (39–214)	43 (23–95)	0.022^¶¶^
**Blood neutrophils ×10^9^·L^−1^**	3.9 (3.6–5.5)	3.6 (2.6–3.9)	0.016^¶¶^
**Prescribed daily dose of ICS µg BDP**	620 (488–1017)	800 (541–925)	0.565^¶¶^
**Dispensed daily dose of ICS µg BDP**	628 (476–983)	301 (90–402)	<0.001^¶¶^
**Daily SABA^#^**	0 (0)	2 (5.7)	0.523^##^
**Daily LABA^#^**	8 (38.1)	10 (28.6)	0.558^##^
**Daily LTRA^#^**	2 (9.5)	2 (5.7)	0.626^##^
**Self-reported use of oral corticosteroid courses for asthma^¶^**	8 (38.1)	4 (11.4)	0.040^##^
**Dispensed oral corticosteroid for asthma per year mg**^**+**^	48 (6–203)	0 (0–99)	0.060^¶¶^
**Co-medications (nonrespiratory)**	0 (0–4)	1 (0–4)	0.472^¶¶^
**AQ20 score**	2 (0–3.5)	2 (1–4)	0.724^¶¶^
**ACT score**	24 (22–25)	24 (22–25)	0.593^¶¶^
**Allergy and/or rhinitis**	14 (66.7)	24 (68.6)	>0.999^##^
**Asthma-related visits to healthcare^¶,§^**	17 (8–27)	9 (6–17)	0.062^¶¶^

Data are presented as n, mean±sd, n (%) or median (interquartile range), unless otherwise stated. BMI: body mass index; BD: bronchodilator; FEV_1_: forced expiratory volume in 1 s; FVC: forced vital capacity; IgE: immunoglobulin E; ICS: inhaled corticosteroid; BDP: beclomethasone dipropionate equivalents; SABA: short-acting β_2_-agonist; LABA: long-acting β_2_-agonist; LTRA: leukotriene receptor antagonist; AQ20: Airways Questionnaire 20; ACT: Asthma Control Test. ^#^: self-reported daily use; ^¶^: examined during the whole 12-year follow-up period; ^+^: dispensed doses of oral corticosteroids (mg) were obtained from the Finnish Social Insurance Institution and were divided by the years of follow-up; ^§^: all respiratory-related scheduled and unscheduled contacts with healthcare due to asthma; ^ƒ^: independent samples t-test; ^##^: Fisher's exact test; ^¶¶^: independent samples Mann–Whitney U-test.

**TABLE 4:** Lung function change (change in pre-bronchodilator forced expiratory volume in 1 s (ΔFEV_1_) from maximum lung function (max_0–2.5_) during the first 2.5 years after diagnosis (after start of therapy) to 12-year follow-up visit) in patients with controlled and not-controlled asthma and different level of adherence (n=181)

	**Good adherence ≥80%**	**Poor adherence <80%**	**p-value**
**Not-controlled asthma n**	125		
ΔFEV_1_ mL·year^–1^	−40 (−56–−20)	−47 (−83–−32)	0.024
ΔFEV_1_ % pred·year^–1^	−0.47 (−0.98–0.25)	−0.76 (−1.40–−0.17)	0.029
**Controlled asthma n**	56		
ΔFEV_1_ mL·year^–1^	−39 (−59–−24)	−35 (−67–−25)	0.859
ΔFEV_1_ % pred·year^–1^	−0.31 (−0.76–0.54)	−0.34 (−1.10–0.07)	0.271

Statistical significances were evaluated by independent-samples Mann–Whitney U-test. When patients with COPD were excluded from the analysis, ΔFEV_1_ was −36 (−54–−18) mL·year^–1^ in patients with >80% adherence and −43 (−78–−28) mL·year^–1^ in patients with <80% adherence and not-controlled asthma (p=0.058).

### Decline in lung function

**FIGURE 3:**
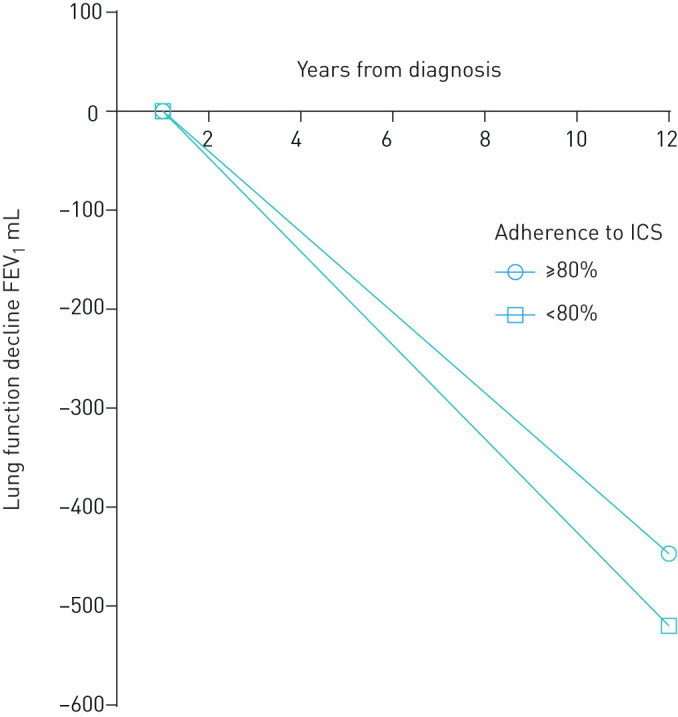
Schematic presentation of the changes in forced expiratory volume in 1 s (FEV_1_) (mL) during 12 years of follow-up in patients with not-controlled asthma and ≥80% or <80% adherence. Model based on group medians. At the year 0 patients were steroid-naïve and inhaled corticosteroid (ICS) treatment was initiated (diagnostic visit). Origin for lung function decline is the maximal point of lung function within 2.5 years after start of treatment.

Next we evaluated the change in lung function in patients with controlled and not-controlled asthma and in groups of ≥80% and <80% 12-year adherence. The patients with not-controlled asthma and <80% 12-year adherence had more rapid decrease in lung function (FEV_1_) compared to patients with ≥80% adherence (p=0.024) (table 4, figure 3). However, no difference was found in patients with controlled asthma between the adherence groups (table 4). We carried out multiple linear regression analysis to find out whether poor adherence predicts accelerated lung function decline in patients with not-controlled asthma when adjusted for age, BMI at follow-up, sex, *F*_eNO_ >20 ppb, ≥10 pack-years and ΔFEV_1_ (baseline−max_0–2.5_) (table 5). After adjustments, poorer adherence (<80%) remained a significant predictor for FEV_1_ (mL) decline.

**TABLE 5:** Predictors for annual decline of forced expiratory volume in 1 s (FEV_1_) (mL) (ΔFEV_1_ from maximum lung function (max_0–2.5_) during the first 2.5 years after diagnosis (after start of therapy) to 12-year follow-up visit) in 12-year follow-up in patients with not-controlled asthma as evaluated by multiple linear regression analysis (n=100)

	**Unstandardised B coefficient (95% CI)**	**p-value**
**Age at follow-up**	−0.10 (−0.53–0.33)	0.638
**Female**	12.46 (1.03–23.89)	0.033
**BMI at follow-up**	−1.03 (−2.05–0.00)	0.049
**≥10 pack-years at follow-up**	−7.92 (−19.01–3.16)	0.159
**ΔFEV_1_ mL** **(baseline**−**max_0–2.5_)**	−0.024 (−0.04–−0.01)	0.005
***F*_eNO_ >20 ppb**	−23.48 (−35.99–−10.97)	<0.001
**Average 12-year adherence (<80%) to ICS**	−10.36 (−20.37–−0.36)	0.042

In univariate analysis, unstandardised B coefficient (95% CI) for average 12-year adherence (<80%) to ICS is −11.23 (−22.15–−0.32), p=0.044. BMI: body mass index; *F*_eNO_: exhaled nitric oxide fraction; ICS: inhaled corticosteroid.

## Discussion

In this study we evaluated both annual and 12-year adherence to ICS from diagnosis to 12-year follow-up visit in patients with adult-onset asthma and different categories of asthma control. The mean adherence to ICS was better in patients with not-controlled than controlled asthma (76% *versus* 63%). Considering patients with not-controlled asthma, good 12-year adherence (≥80%) was associated with daily use of LABA and higher number of peripheral blood neutrophils and asthma-related contacts to healthcare. Importantly, in patients with not-controlled asthma, <80% adherence predicted more rapid lung function decline in adjusted analyses.

Although previous studies have suggested better ICS adherence to be associated with good disease control [4, 11–13, 20], in this study patients with not-controlled asthma had higher 12-year adherence to long-term ICS treatment compared to patients with controlled disease. The higher proportion of adherent patients in the former group may be explained by more severe symptoms and associated need of medication [16, 21]. Conversely, patients with controlled asthma may have themselves stepped-down their ICS therapy after achieving disease control, which would appear as lower adherence rates during the follow-up. In group comparisons, 58% of the patients with not-controlled disease were current or ex-smokers and had significantly more pack-years than those with controlled asthma. This is in line with previous studies which have related smoking to worse asthma control [3, 4, 12, 32]. Furthermore, patients with not-controlled asthma were more often older, male and less often atopic compared to those with controlled disease. In addition, there was a tendency between poorer asthma control and higher BMI. In patients with adult-onset asthma, phenotypes related to obesity and smoking are currently recognised, these phenotypes being at risk of poorer asthma outcomes and disease control [33–35]. Even though patients with not-controlled asthma had mean 12-year ICS adherence as high as 76%, factors such as smoking and obesity may induce insensitivity to ICS and poor response to treatment [34, 36, 37]. Furthermore, in recent studies the average age of patients has been lower in comparison to our study population [10, 13, 14] indicating that previous studies have included more patients with allergic asthma showing predominantly type 2 inflammation. Therefore, ≥80% adherence to long-term ICS treatment appears not to be effective enough to control asthma, since these patients may have had non-type 2 inflammation or untreated comorbidities.

While it seems to have been taken for granted that poor adherence is one common reason behind not-controlled asthma, previous studies in this field have usually been cross-sectional or short-term follow-ups and no long-term studies have been conducted [10–17]. These cross-sectional studies mostly included patients having asthma diagnosis but the information on age of asthma onset, diagnostic criteria or duration of asthma were often lacking [10–12, 14, 16–18]. Moreover, in previous studies asthma control has been defined as asthma symptom control assessed by the Asthma Control Test or Asthma Control Questionnaire and not including both symptoms and lung function as defined by the GINA guideline. Furthermore, adherence has been evaluated with the Medication Adherence Rating Scale or Morisky (Morisky Medication Adherence Scale) questionnaires [4, 12, 14–16, 18]. Such self-reports are widely used for assessing adherence, but may be vulnerable to the shortcomings of these memory-dependent channels. We found one 3-month clinical trial on inhaler adherence in patients with uncontrolled asthma where control was assessed according to GINA guidelines and adherence monitored with an INCA (INhaler Compliance Assessment) device, in which 27% of patients stayed refractory despite being adherent to salmeterol/fluticasone treatment and having correct inhaler technique [38]. A similar result was found in the current study where 34% of the study patients remained not controlled despite having ≥80% adherence to ICS treatment during 12-year follow-up. To our knowledge, this is the first study where asthma control is determined according to GINA guidelines in unselected patient population and adherence is confirmed longitudinally by comparing each patient´s dispensed ICS medication to truly prescribed doses of ICS [8]. Furthermore, all patients with objectively confirmed diagnosis of new-onset adult asthma were included, meaning patients with comorbidities and history of smoking, for example.

When assessing lung function decline during 12-year follow-up in patients with not-controlled asthma, those with lower (<80%) 12-year adherence had more rapid decline in FEV_1_ compared to patients with ≥80% adherence (p=0.024). This difference was not seen in patients with controlled asthma. In addition, the observed difference may be clinically meaningful since the patients with adult-onset asthma rarely remit and a level of 7 mL·year^−1^ would correspond to 140 mL in 20 years and 210 mL in 30 years. Smoking and exacerbations are also important factors associated with the decline in lung function [30]. Even though the two adherence groups with not-controlled asthma did not differ by smoking, we adjusted our analyses for smoked pack-years and found that poorer adherence (<80%) remained a significant predictor for FEV_1_ decline in patients with not-controlled asthma. The finding underscores the importance of determining patients’ asthma control by GINA guidelines and to assess treatment adherence. Early recognition of patients with not-controlled (partially or uncontrolled) asthma and suboptimal (<80%) adherence should allow us to detect those patients who may be at risk of steeper lung function decline in the long term. Moreover, it may allow the opportunity to motivate them towards better adherence and thereby avoid undesirable outcomes in lung function. Conversely, this may help to identify patients whose asthma is not controlled despite high adherence to treatment. These patients may show non-type 2 inflammation, since they have higher blood neutrophil counts and current medications may not be effective enough to control their disease. These results further suggest that patients with late-onset asthma and insufficient therapeutic response need new treatment strategies and possibly other interventions such as support in smoking cessation and weight loss.

In the current study, medical records and pharmacy dispensation data were used in adherence calculations, and therefore some limitations must be addressed. The dispensed medication is not a guarantee of inhaler use, and therefore patient´s adherence to treatment may be overestimated. Although patients had guidance to correct inhaler use when medication was initiated, the correct inhaler technique could not be ensured. Furthermore, asthma control was measured at follow-up visit and not regularly during the follow-up. However, the current study with an exceptionally long follow-up period is based on objectively calculated adherence data, and assessment of asthma control includes lung function according to GINA guidelines [3, 8]. It has been suggested [38] that an assessment of adherence using and electronic device could be beneficial in patients with severe asthma. According to our results such approaches could also be used in patients with not-controlled disease. Future studies should assess how guidance on adherence focused to subjects with poor adherence to ICS and not-controlled asthma affects long-term changes in lung function.

In conclusion, we combined, for the first time, long-term adherence to ICS with asthma control determined according to GINA guideline [27]. The mean 12-year adherence to this treatment was especially high in patients with not-controlled disease. New treatment strategies combining pharmacological and nonpharmacological approaches may be needed in patients with insufficient therapeutic response. Importantly, our results showed that patients with not-controlled asthma and poor adherence (<80%) had more rapid decline in FEV_1_ during 12-year follow-up compared to patients with higher adherence (≥80%), which must be recognised to avoid negative consequences. In clinical practice, careful evaluation of patient´s asthma control and adherence to treatment enhances the recognition of those patients at risk of rapid lung function decline in the long-term.

## Supplementary material

10.1183/23120541.00715-2020.Supp1 **Please note:** supplementary material is not edited by the Editorial Office, and is uploaded as it has been supplied by the author.
Supplementary material 00715-2020.SUPPLEMENT
