# Supplementary material for: Long-term adherence to inhaled corticosteroids and asthma control in adult-onset asthma
Source: ERJ Open Res. 2021 Feb 8;7(1):00715-2020. doi: 10.1183/23120541.00715-2020 (PMC7869602; doi:10.1183/23120541.00715-2020)
Supplement: Supplementary file 1 [file 00715-2020.SUPPLEMENT.pdf]

Long-term adherence to inhaled corticosteroids and asthma control in adult-onset asthma

Iida Vähätalo, Hannu Kankaanranta, Leena E. Tuomisto, Onni Niemelä, Lauri Lehtimäki, Pinja Ilmarinen

Supplementary material

Lung function measurements

Lung function measurements were performed using a spirometer (Vmax Encore 22, Viasys Healthcare, Palm Springs, CA, USA) according to international and national recommendations and Finnish reference values[E1-E3]. Lung function measurement points were: 1) baseline (i.e. time of asthma diagnosis), 2) the maximum lung function (Max0–2.5) during the first 2.5 years after diagnosis (i.e. after start of anti-inflammatory therapy) based on the highest pre-bronchodilator forced expiratory volume in 1 s (FEV1) % pred and 3) after 12 years of follow-up (figure 1). Lung function measurements after the diagnosis of asthma were taken while patients were on medication, without pauses or withholding on the therapy.

Laboratory measurements

Fraction of exhaled nitric oxide (FeNO) was measured with a portable rapid-response chemiluminescent analyzer according to American Thoracic Society standards (flow rate 50 mL·s−1; NIOX System, Aerocrine, Solna, Sweden)[E4]. Venous blood was collected and white

blood cell differential counts were determined. Total immunoglobulin (Ig)E levels were measured by using ImmunoCAP (Thermo Scientific, Uppsala, Sweden)[E4]. Serum levels of IL-6 were determined by ELISA (R & D Systems, Minneapolis, MN, USA) and hsCRP was measured using particle-enhanced immunoturbidometric method on Roche Cobas 8000 automated clinical chemistry analyser (Roche Diagnostics, Basel, Switzerland).

Evaluation of symptoms and dispensed oral corticosteroids

Patients filled out the Airways Questionnaire 20 (AQ20) at baseline visit and during the follow-up visit symptoms were measured both with AQ20[E5] and Asthma Control Test (ACT)[E6]. Dispensed doses of oral corticosteroids (OCS) (mg) were obtained from the Finnish Social Insurance Institution and were divided by the years of follow-up as previously described[E7]. Regarding dispensed OCS, only those having indication for asthma were taken into account.

Asthma control

Patients were separated into two groups by their asthma control at follow-up visit which was defined according to the Global Initiative for Asthma (GINA) 2010 guideline[E8] as previously reported[E9]. Patients with not-controlled asthma (partially or uncontrolled asthma) had at least one of the following features: symptoms of asthma or need for rescue treatment more than twice weekly, decreased lung function (<80% predicted) or limitation of activities due to asthma.

Linear regression analysis

The correlation matrix was analyzed and explanatory variables not strongly correlated ($r<0.7$) (age, gender, BMI, pack years ≥10, ΔFEV_1 (baseline-max0-2.5), average 12-year adherence (<80%) to ICS, FeNO >20 ppb) were included in the analysis. Patients whose FEV_1 annual decline and ΔFEV_1 (baseline-max0-2.5) differed over 2.9-3SD from mean were removed as outliers to ensure homoscedasticity, as well as patients whose age differed over 2.1SD and BMI differed over 2.3SD from mean. We did an additional sensitivity analysis by including also those patients whose age and BMI differed over 2.1SD and 2.3SD from mean and the result regarding adherence remained similar.

Computation of adherence

Prescribed dose for each patient and each year of the follow-up was calculated based on medical records[E7,E10]. All drug and dose changes were taken into account individually for each patient and finally all doses were converted to beclomethasone dipropionate (BDP) equivalents (Example 1)[E10]. Patients´ dispensed doses of ICS were obtained from the Finnish Social Insurance Institution that records all purchased medication from any Finnish pharmacy (Example 1)[E7]. By comparing dispensed doses to prescribed ICS doses, it was possible to evaluate adherence of a single patient during 12-year follow-up period as previously reported[E7]. In the case of ranged doses prescribed e.g. 1-2 puffs 2 times daily we interpreted that patients were adherent when the minimum ICS doses were dispensed. Taking into account, that the renewing of prescription is cost-free and in the case patient continues with the same medication and dosing the prescription is renewed usually for another year (if doctor wants to meet the patient she/he renews smaller amount e.g. 3 months prescription which lasts until the next visit), and therefore there would not be a situation where patient is without prescription. Long-term medication is usually prescribed for 1-2 years in Finland.

The 12-year adherence was calculated by comparing total cumulative dispensed doses of ICS to total cumulative 12-year prescribed doses[E7]. The most commonly used cut-off point (≥80%) in respiratory literature was set also in this study to distinguish the differences between patients with better (≥80%) and poorer (<80%) 12-year adherence[E11-E13]. To obtain a view on the variability of the adherence at long-term follow-up, annual adherence was calculated for each patient individually for each year by dividing patients yearly dispensed ICS doses by yearly prescribed ICS doses (µg BDP equivalents)[E7]. All in all, the extensive 12-year follow-up period and the fact that long-term medication is prescribed continuously, enhanced the evaluation of 12-year ICS adherence including initiation of medication and periods of persistence and temporary non-persistence (Example 2). Moreover, recent publication has used time-varying adherence to describe patient´s adherence behavior and this method was also adapted in the present study[E14]. (Example 2). However, time-varying PDC cannot take into account the dose ranges of asthma medication and therefore we modified the form by using the ug/ug and described the time-varying behavior in year of the follow-up (Example 3). In conclusion, all patients have their individual 12-year time-varying scope of adherence and when combined these together was possible to compare both average 12-year adherence and annual adherence of the patients.

Example 1. 1-year adherence of one example patient.

		SUM
Prescribed doses of ICS (µg) in year 2008	1.1.2008-18.5.2008 (138 days) Pulmicort 200µg 1-2 puffs[£] 2 times a day =138 days*400µg=**55200µg**<br><br>19.5.2008-27.8.2008 (101 days) Pulmicort 400µg 1 puff 2 times a day =101 days*800µg=**80800µg**<br><br>28.8.2008-31.12.2008 (126 days) Symbicort Turbuhaler 200µg/6µg 1-2 doses 2 times a day =126 days*400µg=**50400µg**	**186 400 µg**
Dispensed doses of ICS (µg) in year 2008	Pulmicort Turbuhaler 200µg 1x200 puffs (=one inhaler bought) =200µg*200puffs= **40 000µg**<br><br>Pulmicort Turbuhaler 400µg 1x200 puffs (=one inhaler bought) =400 µg*200 puffs= **80 000µg**<br><br>Symbicort Turbuhaler 200µg/6ug 2x120 puffs (=two inhalers bought) =200 µg*2 inhalers*120puffs= **48000µg**	**168 000µg**
	Adherence = dispensed ICS µg / prescribed ICS µg *100	168 000 / 186 400 =90.1%

[£]In the case of ranged doses prescribed we interpreted that patients were adherent when the minimum prescribed ICS doses were dispensed.

Example 2. 12-year ICS adherence of one example patient.

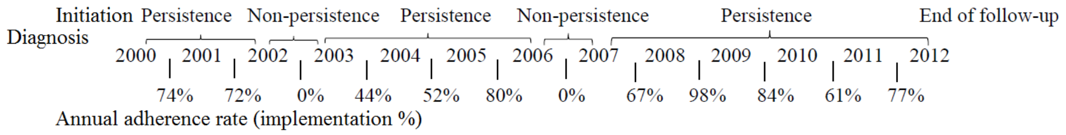

Example 3. Time-varying adherence of one example patient (The average 12-year adherence of the example patient is 68%).

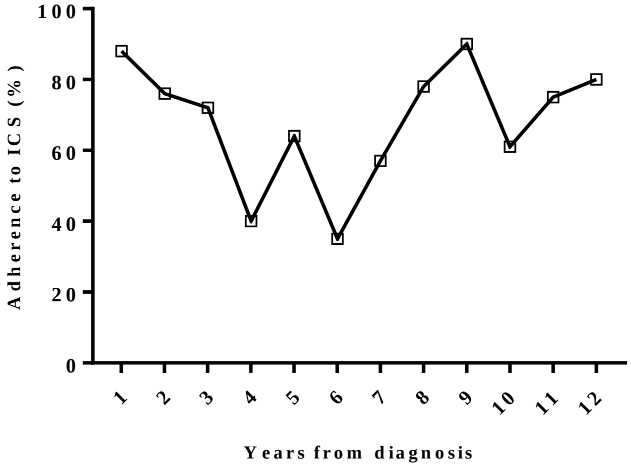

eTable 1. The inclusion and exclusion criteria used in SAAS.

Inclusion criteria	- A diagnosis of new-onset asthma made by a respiratory specialist<br>- Diagnosis confirmed by at least one of the following objective lung function measurements:[a]<br>- FEV_1 reversibility in spirometry of at least 15% and 200 mL after 400 µg of salbutamol<br>- Diurnal variability (⩾20% on at least three days) or repeated reversibility (⩾15%/60 l/min on at least three occasions) during a two-week PEF monitoring<br>- A significant decrease in FEV1 (15%) or PEF (20%) in to exercise or allergen challenge test<br>- A significant reversibility in FEV_1 (at least 15% and 200 mL) or mean PEF (at least 20%) in response to a trial with oral or inhaled glucocorticoids<br>- Symptoms of asthma<br>- Age ≥15 years
Exclusion criteria	- Physical or mental inability to provide signed informed consent<br>- Diagnosis of asthma below the age of 15 years<br>- Of note:<br>- Patients with comorbidities, either other lung disease or any other significant disease, were not excluded<br>- Patients were not excluded because of smoking, alcohol use or any other lifestyle factor<br>- Respiratory symptoms or any other disease during childhood was not a reason to exclude patients, but a diagnosis of asthma at age <15 years was an exclusion criteria

FEV_1= forced expiratory volume in one second, PEF= peak expiratory flow, SAAS= Seinäjoki Adult Asthma Study. Published earlier Kankaanranta et al. 2015[E15]

eTable 2. Characteristics of the study population (n=181).

	Baseline (n=181)	Follow-up (n=181)	p value
Age (y)	47 (13)	59 (13)	<0.001
Female gender n (%)	108 (59.7)	108 (59.7)	
BMI, kg/m^2	27.2 (24.3-30.1)	28.6 (24.5-31.4)	<0.001
Smokers (incl. ex) n (%)	88 (48.6)	91 (50.3)	0.250
Smoking history, pack-y	15 (6.6-21)	18 (7.3-30)	<0.001
Pack-y ≥ 10 and post-BD FEV_1/FVC<0.7 n (%)	15 (8.3)	33 (18.2)	<0.001
Pre-bd FEV_1% pred	81 (70-92)	86 (75-96)	<0.001
Pre-bd FVC % pred	90 (78-100)	97 (87-106)	<0.001
Pre-bd FEV_1/FVC	0.75 (0.68-0.80)	0.73 (0.66-0.79)	<0.001
Post-bd FEV_1% pred	87 (76-98)	89 (80-98)	0.012
Post-bd FVC % pred	94 (82-102)	98 (88-107)	<0.001
Post-bd FEV_1/FVC	0.79 (0.73-0.84)	0.75 (0.68-0.81)	<0.001
Blood eosinophils ($\times10^9 \cdot L^{-1}$)	0.28 (0.17-0.40)	0.18 (0.10-0.28)	<0.001
Total IgE ($kU \cdot L^{-1}$)	84 (36-165)	61 (25-168)	0.187
Daily ICS user n (%)	14 (7.7)	148 (81.8)	<0.001

AQ20 score	7 (4-10)	4 (2-7)	<0.001

Data is presented as n (%), mean (SD) or median (interquartile range). BMI= body mass index, Smoking history, pack-y= pack years of smokers, ICS= inhaled corticosteroid, BD= bronchodilator, FEV_1= forced expiratory volume in 1 second, FVC= forced vital capacity, Daily ICS use= self-reported daily use of ICS, AQ20= airways questionnaire 20. Age is analyzed by paired samples t-test and lung function measurements, inflammatory markers, BMI, pack-years and AQ20 score by related samples Wilcoxon Signed Rank test. Daily ICS users and smokers were analyzed by McNemar test.

eTable 3. Characteristics of patients with not controlled asthma at 12 years after diagnosis according to their level of 12-year adherence (n=125).

	Not-controlled asthma n=125		
	Good adherence (≥80) n=61	Poor adherence (<80) n=64	p-value
**Lung function at follow-up**			
Pre-bd FVC % pred	95 (84-106)	93 (81-101)	0.182[a]
Post-bd FVC % pred	98 (84-108)	95 (84-102)	0.199[a]
FEV_1 Reversibility mL	70 (5-140)	95 (43-178)	0.059[a]
FEV_1 Reversibility % of initial FEV_1	2.7 (0.19-5.2)	3.8 (1.7-8.3)	0.085[a]
**Lung function change**			
ΔFVC mL pred·year^{-1}	-31 (-56 to -8)	-41 (-63 to -14)	0.245[a]
ΔFVC % pred·year^{-1}	0.13 (-0.48 to 0.85)	-0.24 (-0.8 to 0.44)	0.073[a]
ΔFEV_1/FVC·year^{-1}	-0.005 (-0.009 to 0.0)	-0.006 (-0.010 to -0.002)	0.180[a]
**Markers of inflammation**			
FeNO (ppb)	10 (5-15)	10 (5-23)	0.443[a]
IL-6 (pg/mL)	2 (1.2-4.2)	1.9 (1.2-4.2)	0.552[a]
hsCRP	1.2 (0.5-2.4)	1.5 (0.67-3.2)	0.281[a]
**Burden of asthma**			
At least one hospitalization due to asthma n (%)	12 (19.7)	9 (14.1)	0.476[b]
Visits due to acute upper respiratory tract infection or asthma flare-up	6 (2-12)	2 (1-8)	0.053[a]
Asthma control visits	7 (4-12)	6 (3-10)	0.149[a]
**Add-on drugs**			
Daily add-on drug n (%)	49 (80.3)	33 (51.6)	0.001[b]
Daily theophylline n (%)	4 (6.6)	0 (0)	0.054[b]
Daily tiotropium n (%)	5 (8.2)	3 (4.7)	0.485[b]
**Comorbidities**			
Comorbidities (altogether)	1 (0-3)	1 (0-3)	0.487[a]
Treated hypertension	29 (47.5)	18 (28.1)	0.028[b]
Treated dyspepsia	10 (16.4)	3 (4.7)	0.041[b]
Diabetes	11 (18)	10 (15.6)	0.813[b]
Coronary artery disease	9 (14.8)	8 (12.5)	0.797[b]
Depression/Mental health medication	8 (13.1)	8 (12.5)	>0.999[b]
Painful condition	6 (9.8)	8 (12.5)	0.779[b]
**Other**			
Pack-y >=10 and post-BD FEV1/FVC<0.7 n (%)[£]	10 (16.4)	19 (30.2)	0.090[b]
Fulfils severe asthma criteria according to ERS/ATS n (%)	6 (9.8)	5 (7.8)	0.759[b]
Allergy and/or rhinitis n (%)	45 (73.8)	46 (71.9)	0.843[b]
Atopy n (%)[Ω]	14 (26.4)	20 (34.5)	0.413[b]

Data is presented as n (%), mean (SD) or median (interquartile range). FEV_1= forced expiratory volume in 1 second, FVC= forced vital capacity, Lung function change: From max0–2.5 (point of highest lung function during the first 2.5 years after baseline) to 12-year follow-up visit, IL-6= Interleukin 6, hsCRP= High-sensitivity C-reactive Protein, FeNO= fraction of NO

in exhaled air, BD= bronchodilator, Daily add-on drug= self-reported daily use of long-acting β2-agonist, leukotriene receptor antagonist, theophylline or tiotropium, ERS= European Respiratory Society, ATS= American Thoracic Society. Severe asthma was defined according to the ATS/ERS 2014 criteria[E16]. Hospitalizations, asthma control visits and hospital days were examined during the whole 12-year follow-up period. [£]Baseline Pack-y >=10 and post-BD FEV1/FVC<0.7 n (%)= 6 (10) with controlled and 7 (11.3) in patients not-controlled asthma (p= >0.999). [Ω]=atopy was assessed based on skin-prick test. Statistical significances were evaluated by independent samples Mann-Whitney U test ([a]) or by Fisher´s exact test ([b]).

eTable 4. Characteristics of patients with controlled asthma at 12 years after diagnosis according to their level of 12-year adherence (n=56).

	Controlled asthma (n=56)		
	Good adherence (≥80) n=21	Poor adherence (<80) n=35	p-value
**Lung function at follow-up**			
Pre-bd FVC % pred	104 (96-111)	103 (91-110)	0.767[a]
Post-bd FVC % pred	102 (94-109)	102 (92-111)	0.966[a]
FEV_1 Reversibility mL	60 (10-95)	110 (30-160)	0.033[a]
FEV_1 Reversibility % of initial FEV_1	2.7 (0.4-4.6)	3.5 (0.9-6.5)	0.204[a]
**Lung function change**			
ΔFVC mL pred·year^{-1}	-34 (-56 to -13)	-30 (-67 to -11)	0.939[a]
ΔFVC % pred·year^{-1}	0.07 (-0.46 to 0.73)	-0.08 (-0.97 to 0.49)	0.271[a]
ΔFEV_1/FVC·year^{-1}	-0.005 (-0.006 to -0.001)	-0.004 (-0.006 to –0.002)	>0.999[a]
**Markers of inflammation**			
FeNO (ppb)	12 (7-20)	12 (5-16)	0.537[a]
IL-6 (pg·mL^{-1})	1.3 (1.1-2.3)	1.4 (0.91-2.5)	0.735[a]
hsCRP (mg·L^{-1})	0.93 (0.42-1.6)	1.2 (0.47-2.4)	0.441[a]
**Burden of asthma**			
At least one hospitalization due to asthma n (%)	2 (9.5)	4 (11.4)	>0.999[b]
Visits due to acute upper respiratory tract infection or asthma flare-up	5 (0-10)	1 (0-5)	0.152[a]
Asthma control visits	5 (3.5-11.5)	5 (3-7)	0.123[a]
**Add-on drugs**			
Daily add-on drug n (%)	8 (38.1)	11 (31.4)	0.772[b]
Daily theophylline n (%)	0 (0)	0 (0)	
Daily tiotropium n (%)	0 (0)	0 (0)	
**Comorbidities**			
Comorbidities (altogether)	0 (0-2.5)	1 (0-2)	0.857[a]
Treated hypertension	8 (38.1)	10 (28.6)	0.558[b]
Treated dyspepsia	2 (9.5)	0 (0)	0.136[b]
Diabetes	3 (14.3)	4 (11.4)	>0.999[b]
Coronary artery disease	0 (0)	4 (11.4)	0.286[b]
Depression/Mental health medication	4 (19.0)	5 (14.3)	0.715[b]
Painful condition	2 (9.5)	2 (5.7)	0.626[b]
**Other**			
Pack-y >=10 and post-BD FEV1/FVC<0.7 n (%)[£]	2 (9.5)	2 (5.7)	0.626[b]
Fulfils severe asthma criteria according to ERS/ATS n (%)	0 (0)	1 (2.9)	>0.999[b]
Allergy and/or rhinitis n (%)	13 (68.4)	24 (68.6)	>0.999[b]
Atopy n (%)[Ω]	11 (55.0)	16 (48.5)	0.779[b]

Data is presented as n (%), mean (SD) or median (interquartile range). FEV_1= forced expiratory volume in 1 second, FVC= forced vital capacity, Lung function change: From max0–2.5 (point of highest lung function during the first 2.5 years after baseline) to 12-year follow-up visit, IL-6= Interleukin 6, hsCRP= High-sensitivity C-reactive Protein, FeNO= fraction of NO

in exhaled air, BD= bronchodilator, Daily add-on drug= self-reported daily use of long-acting β2-agonist, leukotriene receptor antagonist, theophylline or tiotropium, ERS= European Respiratory Society, ATS= American Thoracic Society. Severe asthma was defined according to the ATS/ERS 2014 criteria[E16]. Hospitalizations, asthma control visits and hospital days were examined during the whole 12-year follow-up period. [£]Baseline Pack-y >=10 and post-BD FEV1/FVC<0.7 n (%)= 0 (0) with controlled and 2 (5.7) in patients not-controlled asthma (p=0.523). [Ω]=atopy was assessed based on skin-prick test. Statistical significances were evaluated by independent samples Mann-Whitney U test ([a]) or by Fisher´s exact test ([b]).

E-supplement references

E1. Viljanen AA, Halttunen PK, Kreus KE, Viljanen BC. Spirometric studies in non-smoking, healthy adults. Scand J Clin Lab Invest Suppl 1982;159:5-20.

E2. Ilmarinen P, Tuomisto LE, Niemelä O, Danielsson J, Haanpää J, Kankaanranta T, et al. Comorbidities and elevated IL-6 associate with negative outcome in adult-onset asthma. Eur Respir J 2016;48:1052-1062.

E3. Tommola M, Ilmarinen P, Tuomisto LE, Haanpää J, Kankaanranta T, Niemelä O, et al. The effect of smoking on lung function: A clinical study on adult-onset asthma. Eur Respir J 2016;48:1298-1306.

E4. American Thoracic Society; European Respiratory Society. ATS/ERS recommendations for standardized procedures for the online and offline measurement of exhaled lower respiratory nitric oxide and nasal nitric oxide, 2005. Am J Respir Crit Care Med 2005;171:912- 930.

E5. Barley EA, Quirk FH, Jones PW. Asthma health status measurement in clinical practice: validity of a new short and simple instrument. Respir Med 1998;92:1207- 1214.

E6. Nathan RA, Sorkness CA, Kosinski M, Schatz M, Li JT, Marcus P, et al. Development of the asthma control test: a survey for assessing asthma control. J Allergy Clin Immunol 2004;113:59- 65.

E7. Vähätalo I, Ilmarinen P, Tuomisto LE, Tommola M, Niemelä O, Lehtimäki L, et al. 12-year adherence to inhaled corticosteroids in adult-onset asthma. ERJ Open Res 2020;6:00324-2019.

E8. Global Initiative for Asthma (GINA). From the global strategy for asthma management and prevention. Updated 2016. Available at: www.ginasthma.org/.

E9. Tuomisto LE, Ilmarinen P, Niemela O, Haanpaa J, Kankaanranta T, Kankaanranta H. A 12-year prognosis of adult-onset asthma: Seinäjoki adult asthma study. Respir Med 2016;117:223-229.

E10. Vähätalo I, Ilmarinen P, Tuomisto LE, Niemelä O, Kankaanranta H. Inhaled corticosteroids and asthma control in adult-onset asthma: 12-year follow-up study. Respir Med 2018;137:70-76.

E11. Papi A, Ryan D, Soriano JB, Chrystyn H, Bjermer L, Rodríguez-Roisin R, et al. Relationship of Inhaled Corticosteroid Adherence to Asthma Exacerbations in Patients with Moderate-to-Severe Asthma. J Allergy Clin Immunol Pract 2018;6:1989-1998.

E12. Souverein PC, Koster ES, Colice G, van Ganse E, Chisholm A, Price D, et al. Inhaled Corticosteroid Adherence Patterns in a Longitudinal Asthma Cohort. J Allergy Clin Immunol Pract 2017;5:448-456.

E13. Engelkes M, Janssens HM, de Jongste JC, Sturkenboom MC, Verhamme KM. Medication adherence and the risk of severe asthma exacerbations: a systematic review. Eur Respir J 2015;45:396-407.

E14. Bijlsma MJ, Janssen F, Hak E. Estimating time-varying drug adherence using electronic records: extending the proportion of days covered (PDC) method. Pharmacoepidemiol Drug Saf 2016;25:325-332.

E15. Kankaanranta H, Ilmarinen P, Kankaanranta T, Tuomisto LE. Seinäjoki adult asthma study (SAAS): A protocol for a 12-year real-life follow-up study of new-onset asthma diagnosed at adult age and treated in primary and specialised care. NPJ Prim Care Respir Med 2015;25:15042.

E16. Chung KF, Wenzel SE, Brozek JL, Bush A, Castro M, Sterk PJ, et al. International ERS/ATS guidelines on definition, evaluation and treatment of severe asthma. Eur Respir J 2014;43:343-373.
